# Autophagy-lysosome inhibitor chloroquine prevents CTLA-4 degradation of T cells and attenuates acute rejection in murine skin and heart transplantation

**DOI:** 10.7150/thno.43507

**Published:** 2020-07-01

**Authors:** Jikai Cui, Jizhang Yu, Heng Xu, Yanqiang Zou, Hao Zhang, Shanshan Chen, Sheng Le, Jing Zhao, Lang Jiang, Jiahong Xia, Jie Wu

**Affiliations:** Department of Cardiovascular Surgery, Union Hospital, Tongji Medical College, Huazhong University of Science and Technology, Wuhan 430022, China.

**Keywords:** Cytotoxic T lymphocyte antigen-4, Chloroquine, Autophagy, T cells, Transplant rejection.

## Abstract

**Background**: The immune checkpoint cytotoxic T lymphocyte antigen-4 (CTLA-4), induced upon T cell activation but degraded quickly, has been targeted in the clinical therapy of advanced cancers and autoimmune diseases. However, whether inhibiting CTLA-4 degradation ameliorates transplant rejection remains unknown.

**Methods**: The CTLA-4 expression in activated murine T cells treated with the inhibitors mediating protein degradation was detected by flow cytometry (FCM). CD45.1 mice, which received TEa T cells and underwent heart transplantation, were administrated with the inhibitor. Subsequently, CTLA-4 expression of TEa T cells was analyzed. Murine skin and heart transplantation models were built, then the survival and histopathology of the allografts, and T cell subsets in the spleens of each group were compared.

**Results**: Chloroquine (CQ) was identified as an inhibitor of CTLA-4 degradation, which augmented both surface and total CTLA-4 expression in T cells. It considerably prolonged the skin and heart allograft survival time and reduced the infiltration of inflammatory cells in allografts. Besides decreasing the frequencies of the CD4^+^ and CD8^+^ effector T cells, especially IFN-γ producing T cells, CQ also increased the proportion of regulatory T cells in the spleen. The CTLA-4 blockade abrogated the benefits of CQ on the survival of heart allografts. Moreover, CQ enhanced CTLA-4 expression in activated human T cells and reduced the secretion of IFN-γ in human mixed lymphocyte reaction.

**Conclusion**: Targeting CTLA-4 degradation provides a novel means to prevent transplant rejection and induce transplant tolerance.

## Introduction

The immune checkpoints, such as programmed cell death protein 1 (PD-1) and cytotoxic T lymphocyte antigen-4 (CTLA-4), are critical modulators of T cell activation and function 1, 2. They are essential for preventing autoimmunity and maintaining immune homeostasis and also play a key role in inducing T cell dysfunction that contributes to tumor immune escape, chronic persistent viral infection, and transplant tolerance 3-5. Our recent study found that enhancing PD-1 expression in T cells by ablating the transcription factor, interferon regulatory factor 4 (IRF4), or inhibiting the MEK1/2 pathway led to T cell dysfunction and induced transplant tolerance in mice 6, 7. However, whether enhancing CTLA-4 expression of alloreactive T cells can prevent transplant rejection remains unknown.

CTLA-4, which down-regulates the immune responses, is constitutively expressed in Tregs andonly induced following T cell activation with the peak levels reaching after 24 - 72 h 8. Expression of CTLA-4 can be regulated not only by many transcription factors, like NFAT and Foxp3, but also by translation and protein degradation 9, 10. Recent research revealed that the T cells of LRBA (lipopolysaccharide-responsive and beige-like anchor protein)-deficient patients undergo accelerated CTLA-4 protein degradation, leading to life-threatening infiltration and autoimmune diseases 11. These results suggest that targeting the degradation of CTLA-4 could be effective in inducing transplant tolerance.

In this study, we screened small-molecule inhibitors, which affect CTLA-4 expression in activated T cells. Chloroquine (CQ) was identified as the inhibitor of CTLA-4 degradation, which augmented both surface and total CTLA-4 expression of T cells *in vitro* and *in vivo*. The CQ treatment considerably prolonged the allograft survival in murine skin and heart transplant models by inhibiting the activation and function of the alloreactive T cells. Moreover, in human CD4^+^ and CD8^+^ T cells, we showed that CQ augmented the CTLA-4 expression and reduced the secretion of IFN-γ in the mixed lymphocyte reaction (MLR). Hence, our findings revealed that inhibiting CTLA-4 degradation could be a novel strategy in transplantation medicine.

## Materials and methods

### Mice

C57BL/6 (B6), BALB/c mice were purchased from Shanghai Model Organisms. TEa TCR transgenic, B6. SJL CD45.1 congenic mice were purchased from Jackson Laboratory (Bar Harbor, MA). The animals were housed in a specific pathogen-free barrier facility at Tongji Medical College, Huazhong University of Science and Technology. All animal experiments were approved by the Institutional Animal Care and Use Committee of Tongji Medical College.

### Murine heterotopic heart transplantation

The murine heart transplantation models were built as described previously 12. Briefly, the hearts harvested from male BALB/c donors (H-2^d^) were transplanted into the abdomens of 8-week-old male B6 or CD45.1 congenic recipient mice (H-2^b^). The recipients were intraperitoneally (i.p.) injected with CQ (800 µg/day, MedChemExpress) or vehicle from the day of transplantation. The treatment continued for a maximum of 14 days. In some experiments, the recipients were i.p. injected with control IgG or anti-CTLA-4 monoclonal antibodies (400 µg/day, clone 9D9, BioXcell) on days 0, 3, and 5. Finger palpation was used to monitor the heart graft survival daily, and rejection was considered to have occurred upon the cessation of the cardiac contractions. Some cardiac allografts were harvested from the recipients on day 7 for further histological analysis. The hematoxylin-eosin (H&E) staining and PR score calculations were performed using a method described previously 13.

Naïve (CD4^+^CD25^-^CD62L^hi^CD44^lo^) TEa T cells (CD45.2^+^ ) 7 were sorted from the splenocytes of the TEa mice using FACSAria flow cytometer, and intravenously (i.v.) injected into CD45.1 congenic mice (2 million per mouse) a day before the surgery. The CD45.1 mice received heart transplantation or sham operation on day 0. On day 4, the mice were i.p. injected with CQ (800 µg) or vehicle, and their spleens were harvested 12 h later and analyzed by flow cytometry (FCM).

### Murine skin transplantation

Ear skin allografts from the male BALB/c mice were transplanted on the back of 8-week-old male B6 mice using a previously described method 14. The recipients were i.p. injected with vehicle or CQ (800 µg/day). The rejection was indicated if the necrotic area of donor skin exceeded eighty percent. Graft rejection level was verified in the skin allografts harvested on day 9 with H&E staining.

### *In vitro* T cell stimulation

The naïve (CD4^+^CD25^-^CD62L^hi^CD44^lo^) T cells were sorted from the spleens of 6-8-week-old male B6 mice with FACSAria flow cytometer. T cells were stimulated with plate-bound anti-CD3e mAbs (5 µg/ml, clone 2C11, BioLegend) and soluble anti-CD28 mAbs (1 µg/ml, clone 37.51, BioLegend). Inhibitors were added to the medium at the following concentrations 15-17: cyclohexane (CHX) (20 µM), CQ (20 µM), bafilomycin A1 (10 µM), 3-MA (50 µM), LY294002 (1 µM), rapamycin (0.5 nM), trichostatin A (200 nM), SAHA (100 nM), and MG-132 (100 µM). T cells were detected at predetermined times with FCM.

### Flow cytometry

The cultured cells and splenocytes were prepared for FCM, as described previously 6. Briefly, extracellular dyeing was performed at room temperature for 10 min. For staining the surface CTLA-4, cells were incubated with antibodies at 37°C for 30 min. The dead cells were excluded using the Zombie Aqua Fixable Viability Kit (BioLegend). Cells were re-stimulated with phorbol 12-myristate 13-acetate (PMA, 50 ng/ml, Sigma-Aldrich) and ionomycin (500 ng/ml, Sigma-Aldrich) and cytokine secretion was blocked with GolgiStop (BD Biosciences) for 4 h according to the manufacturer's instructions. Subsequently, intracellular staining was performed with Foxp3/Transcription Factor Staining Buffer Set (eBiosciences). All samples were processed with the BD LSR Fortessa X-20 flow cytometer, and the results were analyzed using FlowJo v10 software (Tree Star, Inc.). The antibodies used in FCM were as follows: CTLA-4 (UC104B9), CD4 (RM45), CD8 (536.7), CD25 (PC61), CD62L (MEL14), CD44 (IM7), TCR-b (H57597), KLRG1 (MAFA), IFN-γ (XMG1.2), IL17A (TC1118H10), Foxp3 (FJK16s), CD45.1 (A20), CD45.2 (104), CD11b (M170), CD19 (6D5), hFoxp3 (206D), hCTLA-4 (L3D10), hCD4 (OKT4), and hCD8 (RPA-T8).

### Immunoblot analysis

The activated T cells were treated with CQ or PBS for 6 h and then lysed with RIPA lysis buffer (C500005; Sangon Biotech) for 5 min on ice. After centrifuged at 12000g for 5 min at 4°C, the supernatant was prepared for further WB or IP. The following specific antibodies were used in immunoblot analysis: anti-CTLA-4 (ab134090; 1:1000; Abcam), anti-P62 (A5180; 1:1000; Bimake), anti-β-Actin (BA2305; 1:5000; BOSTER).

### *In vitro* human T cell activation and human mixed lymphocyte reaction

Peripheral blood mononuclear cells (PBMCs) were separated with ficoll density gradient centrifugation (P8900; Solarbio) from the peripheral blood of the healthy anonymous donors. Purified CD4^+^ T cells and CD8^+^ T cells were activated with anti-CD3/CD28 mAb-coated beads (bead:cell = 1:1, Dynabeads, Invitrogen), IL-2 (100 U/ml, Peprotech), and CQ (20 µM) or PBS were added to the medium. On day 1, the cells were collected for FCM or PCR. For the human MLR 18, sorted CD14^+^ monocytes were cultured with GM-CSF (1000 U/ml; Peprotech) and IL-4 (1000 U/ml; Peprotech) for 5 days and stimulated with LPS (5 ng/ml; Sigma-Aldrich) for 2 days. Next, the sorted CD4^+^ T cells were incubated with allogeneic dendritic cells for 6 days. And ipilimumab (1 µg/ml, Topscience), CQ (20 µM), or PBS were added to the medium on day 0 and 4. The concentration of IFN-γ in the supernatant was detected using ELISA kits (RK00015; ABclonal).

### Statistical analysis

Data are presented as the mean ± SD. The *p*-values were analyzed with the unpaired Student's t-test or Mann-Whitney test by GraphPad Prism Software (version 7.0a). Statistical significance was set as *p* < 0.05 and expressed as *.

## Results

### CQ prevents CTLA-4 degradation of T cells upon activation *in vitro*

**Figure 1:**
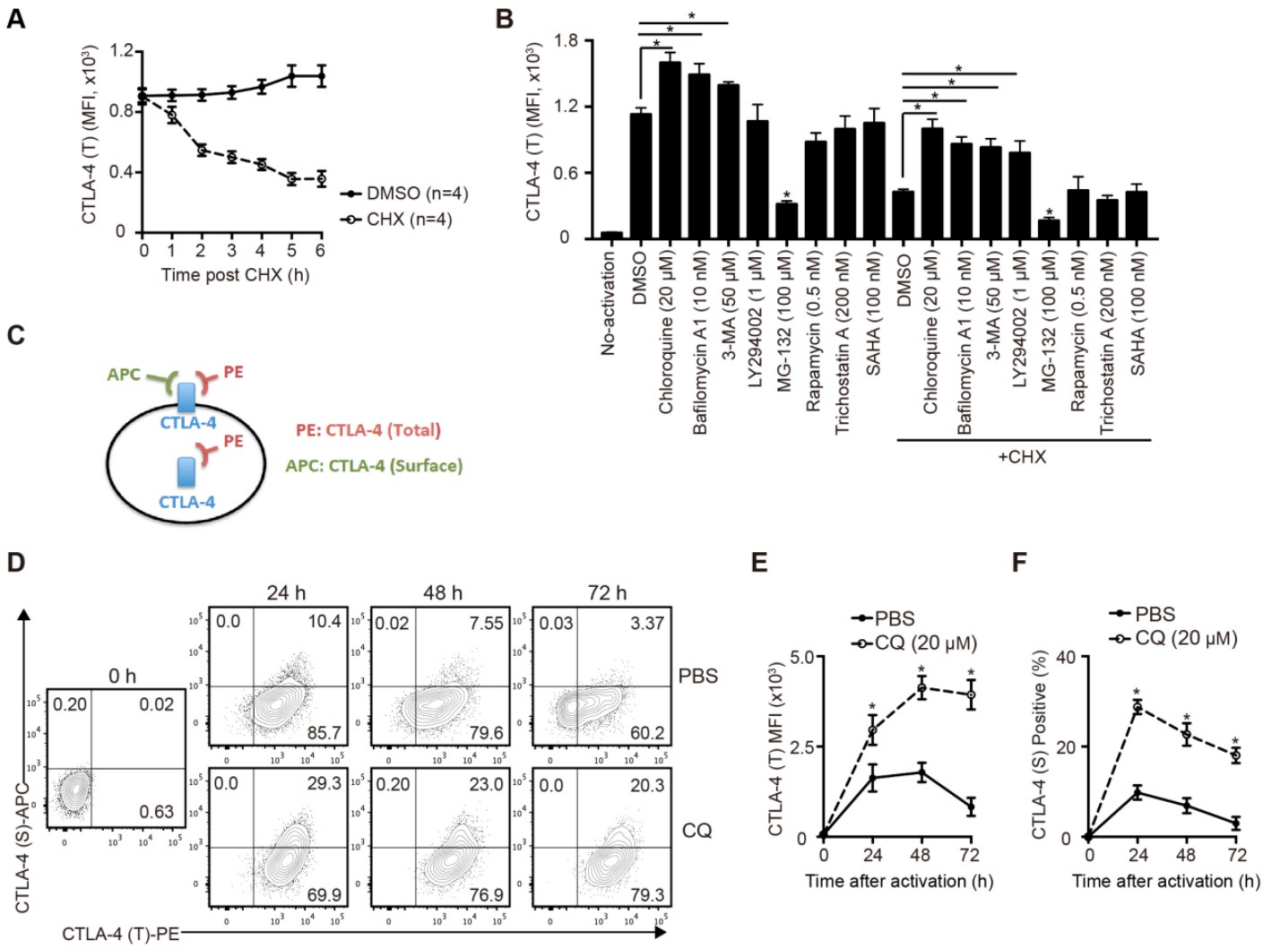
** CQ prevents CTLA-4 degradation of T cells *in vitro*.** (A) The total amount of CTLA-4 protein was assessed by FCM. (B) Activated T cells were treated with different inhibitors for 6 h, with or without CHX, and then collected for FCM. (C) Schematic representation of the CTLA-4 staining method. (D) Expression of CTLA-4 (S) and CTLA-4 (T) was detected by FCM. (E, F) Time-dependent MFI of CTLA-4 (T) and percentage of CTLA-4 (S)^+^ T cells after activation. Data are expressed as the mean ± SD and representative of three independent experiments. *p<0.05; unpaired Student's test.

To study the degradation of CTLA-4 protein of murine T cells, we sorted the naïve CD4^+^ T cells from B6 mice spleens and activated them for 24 hours *in vitro*. After blocking protein synthesis with CHX, we analyzed the time-dependent changes of CTLA-4 expression by FCM. The results showed that more than half of CTLA-4 was degraded quickly within 6 h (Figure 1A). To identify the degradation pathway of CTLA-4, we performed an inhibitor screening assay by treating the activated T cells with inhibitors that mediated protein degradation with or without CHX. We found that the autophagy inhibitors, such as CQ and bafilomycin A1, dramatically decreased the degradation of CTLA-4, while the proteasome inhibitor MG-132 accelerated its degradation (Figure 1B). To verify the specific degradation pathways of CTLA-4, we analyzed the proteins levels of CTLA-4 and autophagy marker P62 in activated T cells with immunoblot and found that CQ treatment inhibited autophagy and increased CTLA-4 protein level at the same time (Figure S1A). Moreover, endogenous co-immunoprecipitation in murine T cell lysate showed CTLA-4 could combine with P62 (Figure S1B), which usually mediates the selective autophagic degradation of ubiquitinated proteins 19, indicating that CTLA-4 is degraded through the autophagic lysosomal pathway. Since CTLA-4 functions on the cell surface, we adopted a staining method to distinguish the surface (functional) CTLA-4 (S) from the total CTLA-4 (T) (Figure 1C). Also, to investigate the overall effect of CQ on CTLA-4 expression, we treated the T cells with CQ at the beginning of the activation and used FCM to detect surface and total CTLA-4 expression at 24, 48, and 72 h (Figure 1D). Figures 1E and F show that CQ treatment not only led to higher mean fluorescence intensity (MFI) of CTLA-4 (T) but also increased the frequency of CTLA-4 (S)-positive cells. These results indicated that the autophagy inhibitor CQ could prevent CTLA-4 degradation of the activated T cells.

### CQ augments CTLA-4 expression of alloreactive T cells *in vivo*

**Figure 2:**
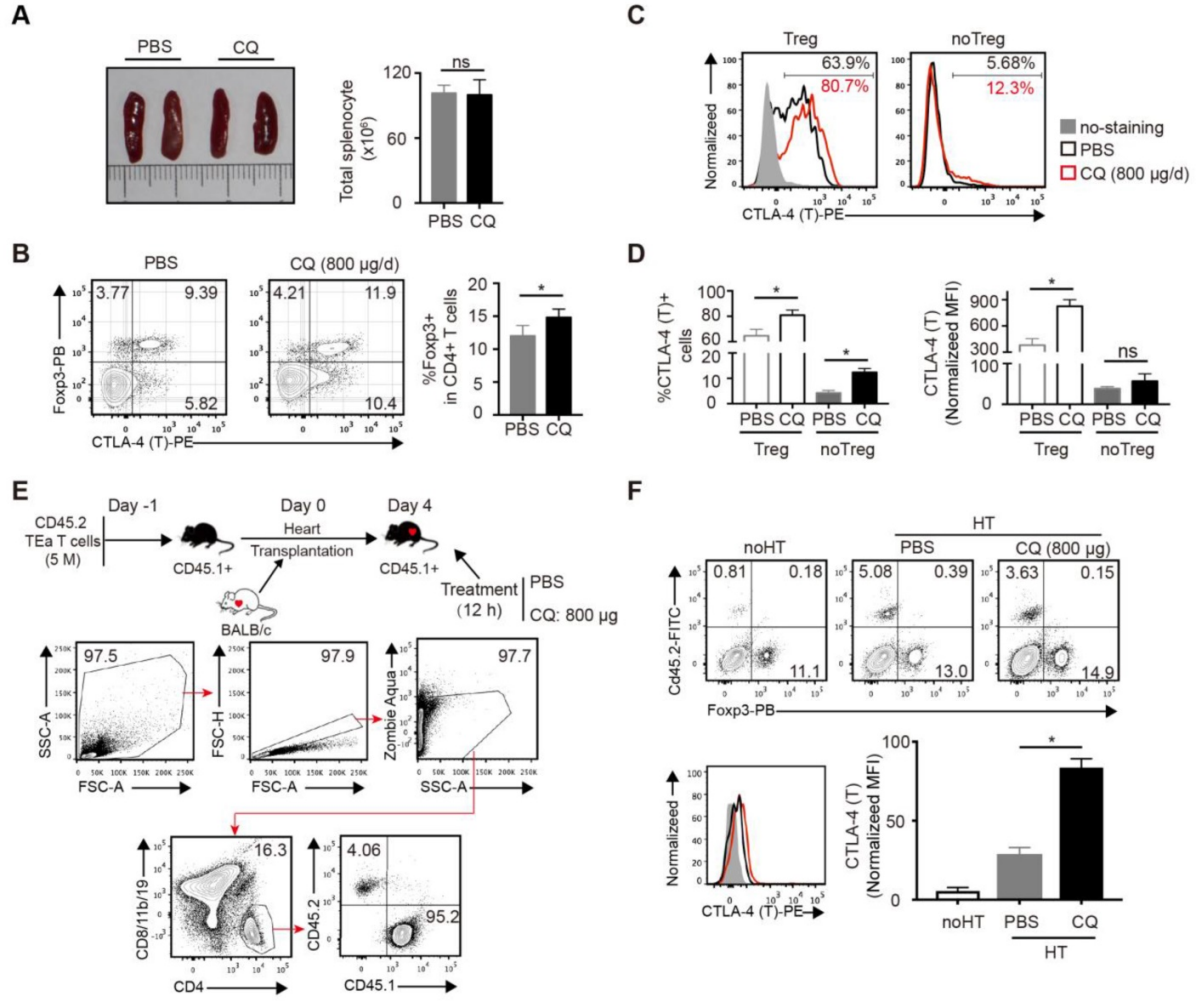
** CQ augments CTLA-4 expression of T cells *in vivo*.** (A) Representative images of spleens and quantification of total numbers of splenocytes of two groups. (B) FCM showed the percentage of Tregs among CD4^+^ T cells. (C, D) Percentages of CTLA-4^+^ cells and CTLA-4 MFI among Tregs and no Tregs. The relative CTLA-4 level was normalized to fluorescence minus (FMO) control. (E) Schematic representation of the experimental design. FCM plots show the gating strategy to detect the transferred TEa T cells. (F) FCM plots show the percentage of CD45.2^+^ TEa T cells of the two groups. Histograms display CTLA-4 MFI of TEa T cells. Data are presented as the mean ± SD. *p<0.05; unpaired Student's test. Data are the mean ± SD and representative of three independent experiments with 5 mice in each group.

Next, we studied the effect of CQ *in vivo*. Based on a previous study 20 and a CQ dose-escalation assay, we determined that 800 µg/day CQ was the best dose, which had no influence on the survival rates and weight of mice (Figure S2A-B). To assess the influence of CQ on the immune system, B6 mice were treated with CQ or PBS i.p. for 5 days, following which the spleens were harvested and analyzed. Compared to the control group, the size of spleens and the total number of splenocytes showed little difference in the CQ treatment group (Figure 2A), but the percentage of Tregs (CD4^+^FOXP3^+^) among CD4^+^ T cells increased (Figure 2B). Furthermore, both the proportion of CTLA-4^+^ cells and normalized MFI of CTLA-4 showed a significant upregulation in the Tregs from the CQ-treated mice. In the non-Tregs (CD4^+^FOXP3^-^), CQ administration led to a higher proportion of CTLA-4^+^ cells (Figure 2C-D). Since the TEa T cells are only activated via recognizing the BALB/c I-Eα allopeptide presented by B6 APCs 21, we established the TEa T cell transfer and heart transplant model to identify the effect of CQ on alloreactive T cells. As displayed in Figure 2E, gating of the TEa T cells (CD4^+^CD8/11b/19^-^CD45.2^+^) demonstrated that CQ administration led to a noticeable upregulation in the total CTLA-4 expression among alloreactive T cells (Figure 2F). These results suggested that CQ could augment CTLA-4 expression of alloreactive T cells *in vivo*.

### CQ protects acute allograft rejection in mouse skin and heart transplant models

**Figure 3:**
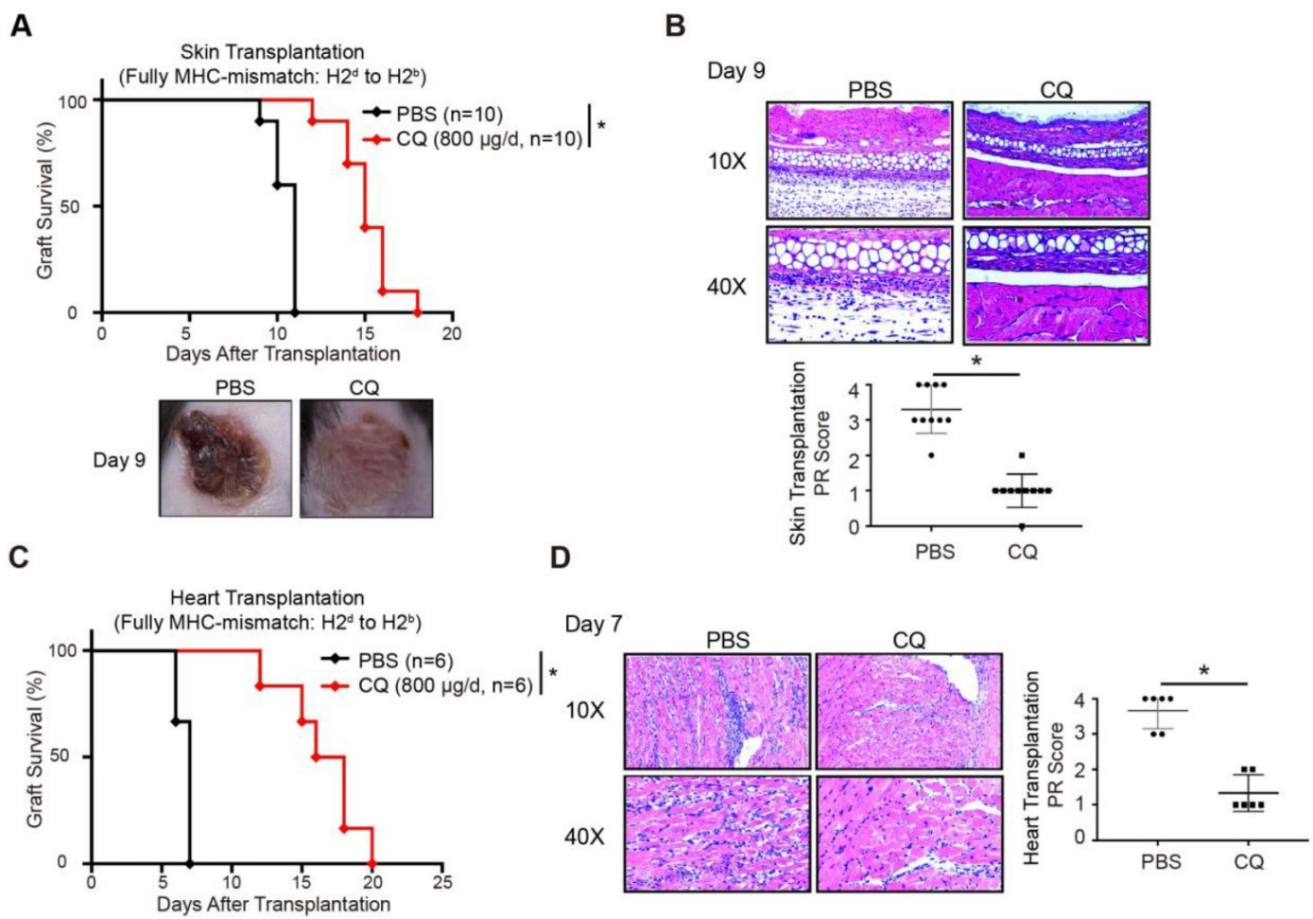
** CQ protects acute allograft rejection in mouse skin and heart transplant models.** (A) Proportion of skin graft survival with time after transplantation (n=10). Representative images of skin grafts in different groups on day 9. (B) Representative images of H&E-stained sections of skin grafts harvested from both groups on day 9, along with the PR scores. (C) Percentage of heart graft survival with time after transplantation (n=6). (D) Representative images of H&E-stained sections of heart grafts harvested from both groups on day 7, along with the PR scores. Ten graft sections per mouse and 10 or 6 mice were analyzed in every group, and two representative images (10× and 40×) per group are displayed. *p<0.05; Mann-Whitney test. Data are the mean ± SD and representative of three independent experiments.

A previous study showed that enhanced CTLA-4 expression might contribute to transplantation tolerance 22. To explore the influence of CQ on acute allograft rejection, we established murine skin transplant models and treated the recipients with CQ or PBS. Compared to the PBS group, CQ administration prolonged the allograft survival time (CQ MST = 15.10 ± 0.50 days, n = 10; PBS MST = 10.50 ± 0.22 days, n = 10) (Figure 3A). The H&E staining displayed that allografts harvested from the CQ group on day 9 contained less inflammatory infiltration and necrosis than the PBS group, consistent with a significantly lower PR score (Figure 3B). Subsequently, we studied the impact of CQ in the heart transplant model. Our data showed that CQ treatment substantially prolonged cardiac allograft survival compared to the PBS group (CQ MST = 16.50 ± 1.15 days, n = 6; PBS MST = 6.67 ± 0.21 days, n = 6) (Figure 3C). The allograft harvested from the CQ group contained less inflammatory infiltration in the myocardium, necrotic cardiomyocytes, and vasculopathy and a significantly lower PR score (Figure 3D). These results demonstrated that CQ attenuates acute allograft rejection in murine skin and heart transplant models.

### Impacts of CQ on the subsets of T cells in the spleen after heart transplantation

**Figure 4:**
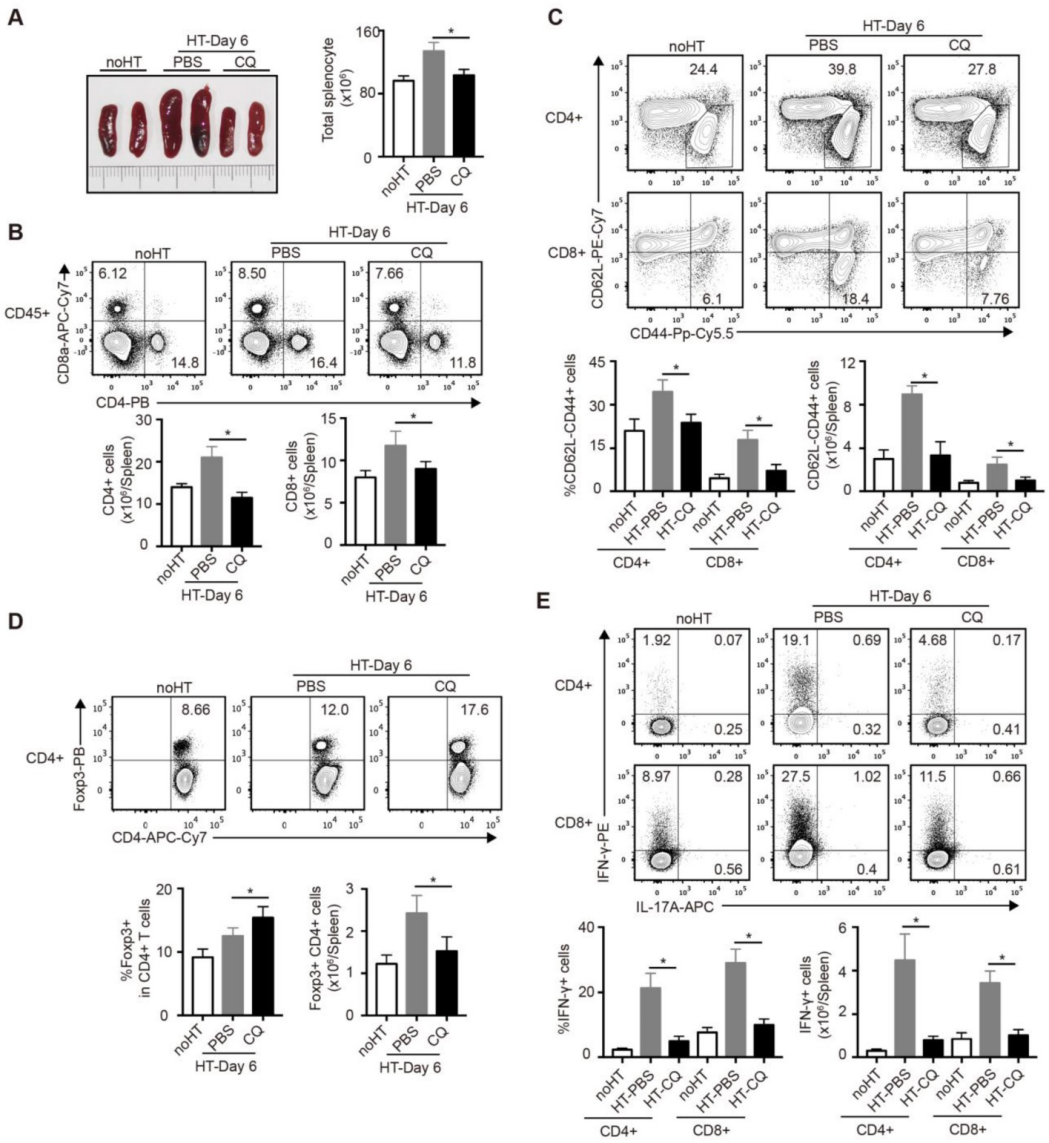
** Impacts of CQ on the subsets of T cells in the spleen after heart transplantation.** (A) Representative images of the spleens (two pictures/group) and total numbers of splenocytes were manually calculated. (B) FCM analysis of the proportions and numbers of CD4^+^ T cells and CD8^+^ T cells from spleens harvested from each group. (C) FCM plots and histograms show the percentage of effector (CD62L^lo^CD44^high^) CD4^+^ and CD8^+^ T cells in each group. (D) FCM plots and histograms show the ratios of Tregs (Foxp3^+^) in CD4^+^ T cells and the number of the Foxp3^+^CD4^+^ T cells. (E) FCM analysis of the percentage of CD4^+^ and CD8^+^ T cells secreting IFN-γ or IL-17A. Data are the mean ± SD and representative of three independent experiments (n=6). *p<0.05; unpaired Student's test.

To further investigate the influence of CQ on the immunoreaction after heart transplantation, we harvested and analyzed the spleens of cardiac allograft recipients on day 6. As shown in Figure 4A, the PBS group developed splenomegaly and showed increased spleen cell numbers, whereas the CQ group showed little difference in the size and cell number of spleens compared to the group without heart transplantation. Next, we used FCM to examine the number and percentage of T cell subpopulation in the spleen. The proportion and number of total CD4^+^ and CD8^+^ T cells after CQ treatment were dramatically lower than in the control group (Figure 4B). Furthermore, CQ treatment decreased the proportion and number of the effector (CD62L^lo^CD44^hi^) CD4^+^ and CD8^+^ T cells (Figure 4C). In contrast, the ratio of Tregs among CD4^+^ T cells increased after CQ treatment, although the number of Tregs per spleen was lower than that of the PBS group (Figure 4D). Besides, CQ administration led to lower frequencies of IFN-γ-producing cells within both CD4^+^ T and CD8^+^ T cells (Figure 4E). Overall, CQ played an inhibitory role in T cell activation and function during heart transplantation.

### Prolonged allograft survival in mice treated with CQ is CTLA-4 dependent

**Figure 5:**
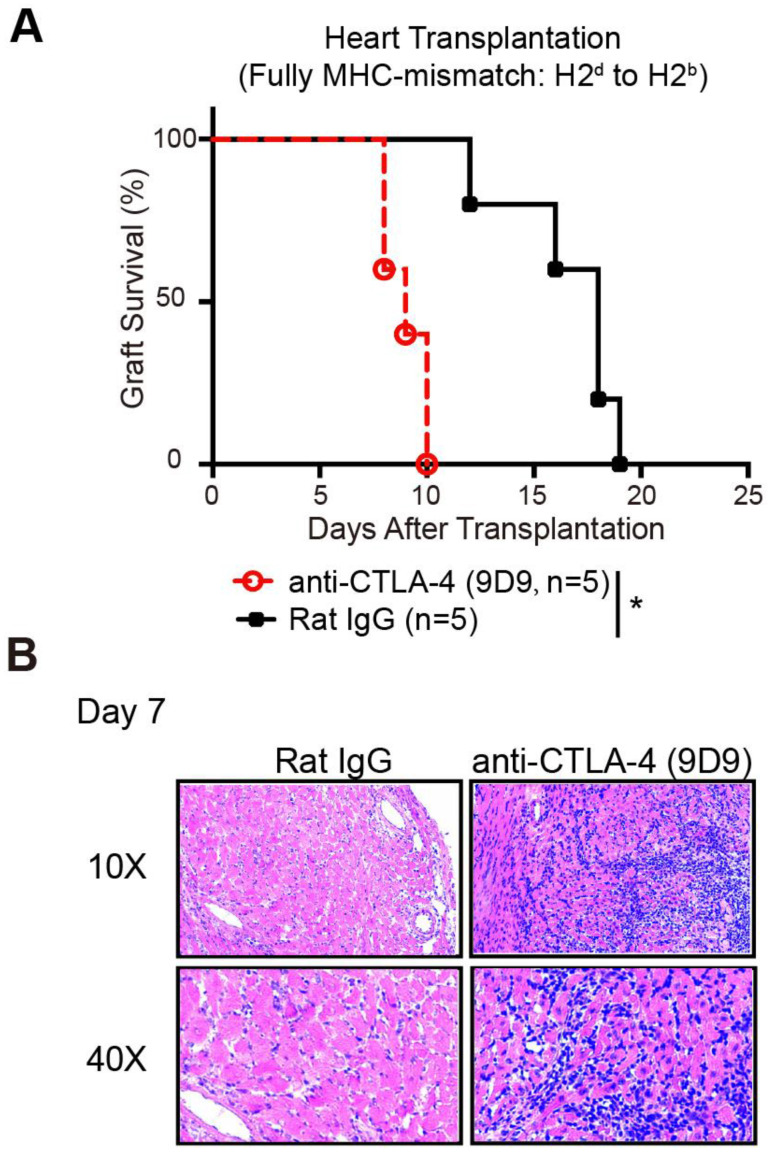
** Prolonged allograft survival in mice treated with CQ is CTLA-4 dependent.** (A) Percentage of heart graft survival with time after transplantation (n=5). (B) Representative images of H&E stained sections of heart grafts harvested on day 7, along with the PR scores. Ten graft sections per mouse and 5 mice in every group were analyzed, and two representative images (10× and 40×) per group are displayed. *p<0.05; Mann-Whitney test. Data are the mean ± SD and representative of three independent experiments.

To confirm whether the protective role of CQ in allograft survival is the outcome of enhanced CTLA-4 expression, we established murine heart transplant models. We treated the recipients with PBS plus anti-CTLA-4 mAbs, CQ plus anti-CTLA-4 mAbs, or CQ plus control IgG. The results showed that blocking the CTLA-4 pathway abolished the benefits of CQ on the survival of heart allografts (CQ plus control IgG MST = 16.60 ± 1.25 days, n = 5; CQ plus anti-CTLA-4 MST = 9.00 ± 0.25 days, n = 5; PBS plus anti-CTLA-4 MST = 7.60 ± 0.89 days, n = 5) (Figure 5A). Consistent with this observation, histological analysis demonstrated that inflammatory infiltration and necrosis in the myocardium increased dramatically after the neutralization of CTLA-4 (Figure 5B). Thus, we could conclude that the prolonged allograft survival in the CQ treatment group was CTLA-4 dependent.

### CQ enhances CTLA-4 expression of human CD4^+^ and CD8^+^ T cells and reduces IFN-γ secretion in human MLR

**Figure 6:**
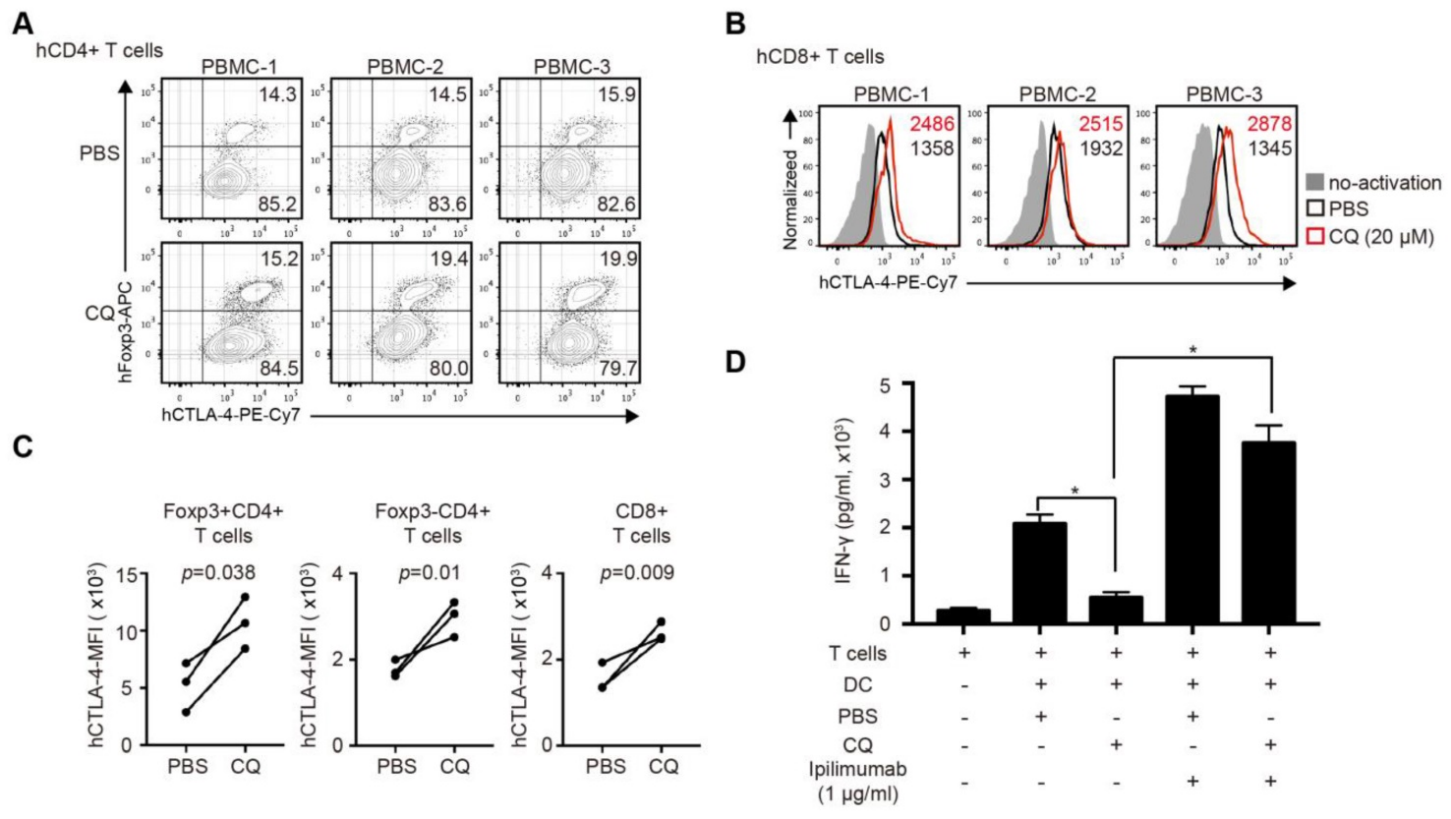
** CQ regulates CTLA-4 protein turnover of human CD4^+^ and CD8^+^ T cells and reduces the secretion of IFN-γ in human mixed lymphocyte reaction.** (A) Percentage of Foxp3^+^CTLA-4^+^ T cells in three samples. (B) Histograms show the expression of CTLA-4 among CD8^+^ T cells in three samples after different treatments. (C) Quantification of CTLA-4 MFI of Foxp3^+^CD4^-^ T cells, Foxp3^-^CD4^+^ T cells, and CD8^+^ T cells in three samples after different treatments. (D) Concentration of IFN-γ by ELISA in the supernatant of MLR; histogram shows the concentration of IFN-γ after different treatments. Data of ELISA are the mean ± SD and representative of three independent experiments. *p<0.05; unpaired Student's test.

The clinical relevance of CQ was further investigated in human T cells. We activated purified human CD4^+^ and CD8^+^ T cells for 24 hours with the addition of CQ or PBS. The percentage of Tregs in CD4^+^ T cells in the CQ group was higher than that in the PBS group (Figure 6A). The mRNA level of Ctla-4 showed no difference in two groups (Figure S3A-B), while CQ administration led to a higher level of CTLA-4 MFI in Foxp3^+^CD4^+^ T cells, Foxp3^-^ CD4^+^ T cells, and CD8^+^ T cells (Figure 6B-C). Also, to evaluate the impact of CQ on the secretion of T cell inflammatory factor, we performed human MLR and collected the supernatant on day 6. The ELISA assay demonstrated that the concentration of IFN-γ was significantly lower in the CQ group compared to the PBS group, which was abrogated by adding ipilimumab (Figure 6D). Therefore, we could infer that CQ could augment the CTLA-4 expression of human T cells.

## Discussion

In our study, the autophagy inhibitor CQ enhanced CTLA-4 expression of T cells *in vitro* and *in vivo* and prolonged murine skin and heart allograft survival by inhibiting the activation and function of alloreactive T cells. Furthermore, CQ also augmented the CTLA-4 expression of human T cells and reduced the secretion of IFN-γ in the human MLR. Hence, our findings indicated that inhibiting CTLA-4 degradation might be a therapeutically relevant strategy in transplantation medicine.

It has been reported that some transplant recipients treated with ipilimumab to treat malignant melanoma developed graft failure 23-25, highlighting the significance of CTLA-4 in the maintenance of transplant tolerance. Although CTLA-4 Ig (Belatacept) has been approved for the treatment of transplant recipients 26, the application has been limited because of significant side effects 27. One possible reason could be its unselective inhibition of CTLA-4 signaling 28. Therefore, augmenting the endogenous expression of CTLA-4 could be a promising strategy to overcome this problem 18. In T cells, over 90% of CTLA-4 is present mainly in the intracellular vesicles trafficking to the plasma membrane upon T cell activation 29. Hence, a staining method was adopted to distinguish functionally active surface CTLA-4 from total CTLA-4 *in vitro*. However, *in vivo*, the distinction was not possible because of the limited amount of surface CTLA-4 and the difficulty in accurately capturing the activation time of T cells. Several CTLA-4 antibodies were employed, but none of them could effectively display the surface CTLA-4 (data not shown). Still, we provided evidence that the change of surface CTLA-4 was consistent with total CTLA-4 (Figure 1D), and the augmented total CTLA-4 expression was functional *in vivo*.

Considering the dynamic characteristics of CTLA-4, we inferred that restraining the degradation rather than promoting its synthesis tended to enhance the functional CTLA-4 expression on the plasma membrane more efficiently. Therefore, an inhibitor screening assay was performed with various inhibitors of protein degradation. The analysis revealed that inhibiting lysosome activity, but not proteasome activity, substantially rescued the CTLA-4 protein levels. Notably, the proteasome inhibitor MG-132 dramatically accelerated the degradation of CTLA-4, possibly because the proteasome pathway's inhibition promoted the autophagy-lysosome pathway instead. Among the inhibitors we screened, CQ showed the most potent effect in enhancing the CTLA-4 expression, with or without CHX. Although its effect on both the transcriptional and translational levels of CTLA-4 expression could not be excluded. Moreover, the molecular mechanism of the CTLA-4 degradation pathway is still unclear and warrants further research.

As an autophagy inhibitor 30, CQ has been used as an antimalarial drug for decades 31. Recently, it has occasionally been used in controlling rheumatoid arthritis and lupus erythematosus 32, but the underlying mechanism has not been determined to date. Previous research indicated that CQ might suppress inflammation by enhancing glucocorticoid activity in macrophages 20. However, no information was available on the possible effects of CQ on T cells 33. Our present study revealed that the augmented CTLA-4 pathway might account for the clinical benefits of CQ treatment. CQ might also impact other immune cells, like macrophages, because, compared to PBS plus anti-CTLA-4, CQ plus anti-CTLA-4 slightly prolonged the allografts survival time, but with no statistical significance. Furthermore, although the CTLA-4-blocking antibody abrogated the beneficial effects of CQ, the outcome might result from a more global role in regulating the immune responsiveness of CTLA-4 28. Indeed, we couldn't exclude the influence of cellular autophagy due to the inhibitor specificity. To address this issue, the detailed mechanisms of CTLA-4 degradation need to be determined, so that a target that inhibiting CTLA-4 degradation without affecting cellular autophagy could be reached.

Given the opposite effects of CQ and rapamycin on CTLA-4 expression and the synergistic effect of CTLA-4-Ig and rapamycin on the protection of transplantation immunity 34, we used rapamycin and CQ in combination. Indeed, the MFI of total CTLA-4 in T cells after the administration of CQ plus rapamycin was higher than rapamycin treatment alone (Figure S4A). Compared to the rapamycin group, CQ plus rapamycin administration moderately prolonged the allograft survival time (CQ plus rapamycin MST = 27.00 ± 1.77 days, n = 6; rapamycin MST = 15.83 ± 1.20 days, n = 6; Figure S4B). Thus, CQ could enhance the protective effect of rapamycin on the allograft. However, the synergistic effect is moderate. We infer it relates to the extensive effects of CQ conflicting with rapamycin in some fields. Finding a target that specifically inhibiting CTLA-4 degradation will possibly contribute to improve this issue. Besides, the respective dosage of CQ and rapamycin need further exploration to reach the maximum effect. Moreover, finding a suitable drug carrier might contribute to maximize the effect 35.

In summary, this study revealed that CTLA-4 is degraded through the autophagy-lysosome pathway. CQ, as an autophagy inhibitor, could augment CTLA-4 expression upon T cell activation, both *in vitro* and *in vivo*, and prolong murine skin and heart allograft survival. Moreover, CQ could regulate CTLA-4 protein turnover of human CD4^+^ and CD8^+^ T cells and reduce the secretion of IFN-γ in the human MLR. Hence, our findings indicated that inhibiting CTLA-4 degradation might be a therapeutically relevant strategy in transplantation medicine. Further work is needed to study the detailed molecular mechanisms of the CTLA-4 degradation pathway.

## Supplementary Material

Supplementary figures and tables.

## Abbreviations

CTLA-4: totoxic T-lymphocyte Antigen 4
CQ: Chloroquine
MLR: Mixed Lymphocyte Reaction
Tregs: Regulatory T cells
IFN-γ: Interferon Gamma
PD-1: Programmed Cell Death Protein 1
IRF4: Interferon Regulatory Factor 4
i.p.: Intraperitoneally
i.v.: Intravenously
H&E: Hematoxylin & Eosin Staining
PR: Parenchymal rejection
FCM: Flow Cytometry
PBMC: Peripheral Blood Mononuclear Cells
ELISA: Enzyme-linked Immunosorbent Assay
APCs: Antigen-Presenting Cells
GM-CSF: Granulocyte-Macrophage Colony-Stimulating Factor
CHX: Cyclohexane
MFI: Median Fluorescence Intensity
MST: Mean Survival Time
