# Autophagy-lysosome inhibitor chloroquine prevents CTLA-4 degradation of T cells and attenuates acute rejection in murine skin and heart transplantation: Erratum

**DOI:** 10.7150/thno.73353

**Published:** 2022-04-26

**Authors:** Jikai Cui, Jizhang Yu, Heng Xu, Yanqiang Zou, Hao Zhang, Shanshan Chen, Sheng Le, Jing Zhao, Lang Jiang, Jiahong Xia, Jie Wu

**Affiliations:** Department of Cardiovascular Surgery, Union Hospital, Tongji Medical College, Huazhong University of Science and Technology, Wuhan 430022, China.

The original publication of this article unfortunately contained an inadvertent mistake in Figure 6A, which has been corrected in the figure below. In Figure 6A of the original article, the scatter diagram of PBMC-3 group under PBS treatment has a copy-paste error from PBMC-2 group under PBS treatment, which was caused during the preparation of figures. The authors confirm that the corrections made in this erratum do not affect the original conclusions. The authors apologize for any inconvenience or misunderstanding that this error may have caused.

## Figures and Tables

**Figure 6:**
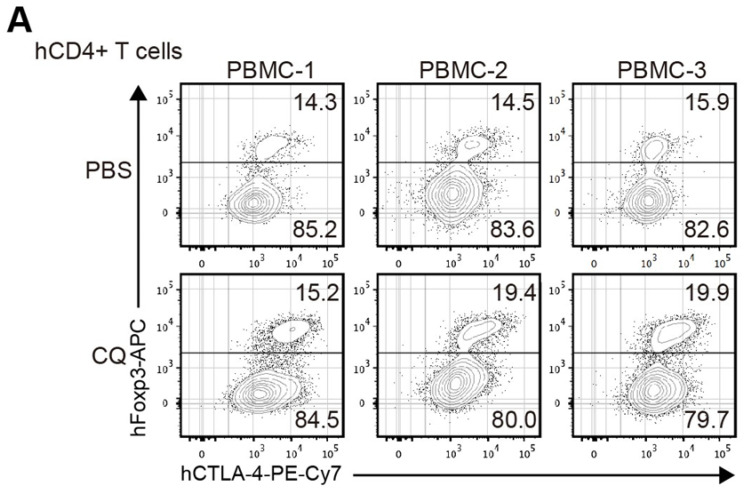
A. Corrected image for original Figure 6A.

