# Supplementary material for: Autophagy-lysosome inhibitor chloroquine prevents CTLA-4 degradation of T cells and attenuates acute rejection in murine skin and heart transplantation
Source: Theranostics. 2020 Jul 1;10(18):8051–60. doi: 10.7150/thno.43507 (PMC7381746; doi:10.7150/thno.43507)
Supplement: Supplementary file 1 — Supplementary figures and tables. [file thnov10p8051s1.pdf]

Figure S1. CQ inhibited the autophagic lysosomal degradation of CTLA-4 in T cells. (A) Sorted T cells were activated with anti-CD3/28 for 12 h and then treated with CQ or PBS for 6 h before collected to detect the expression of CTLA-4 and P62 with immunoblot analysis. (B) Endogenous co-IP of CTLA-4 and P62 in murine T cell lysate. Naïve $CD4^+$ T cells were obtained by FACS and activated with anti-CD3/CD28 antibodies for 24 h before lysis. Antibodies against CTLA-4 were used to pull down target proteins and their interaction partners, which were resolved under denaturing conditions and visualized by immunoblotting. Data are representative of three independent experiments.

Figure S2. Impact of Chloroquine dose escalation on survival and weight in mice. 8-week-old C57 male mice were randomly divided into 5 groups (8 mice in each group) and i.p. with different doses of CQ (400ug/d, 800ug/d, 1200ug/d, 1600ug/d) or PBS for 14 days. (A) Survival rates were calculated. (B) Weight of mice was recorded, and the ratio of weight after treatment and original weight was calculated. Data are the mean ± SD and representative of three independent experiments. *$p<0.05$; unpaired Student's test.

Figure S3. CQ does not influence Ctla-4 mRNA expression of human $CD4^+$ and $CD8^+$ T cells. (A) Histograms show the relative expression of CTLA-4 among $CD4^+$ cells in three samples after different treatments by quantitative reverse transcriptase-PCR (qRT-PCR). (B) Histograms show the relative expression of CTLA-4 among $CD8^+$ cells in three samples after

different treatments by quantitative reverse transcriptase-PCR (qRT-PCR). Data are the mean ± SD and representative of three independent experiments. *p<0.05; unpaired Student's test.

Figure S4. Effect of combined Chloroquine and Rapamycin treatment on allograft survival. (A) Sorted T cells were activated with anti-CD3/28 for 12 h, and then treated with CQ, rapamycin, CQ plus rapamycin or vehicle for 6 h before analyzed by FCM. Histogram shows CTLA-4 MFI of T cells after different treatments. (B) Percentage of heart graft survival with time after transplantation (n=6). Data are the mean ± SD and representative of three independent experiments. *p<0.05; unpaired Student's test or Mann-Whitney test.

A

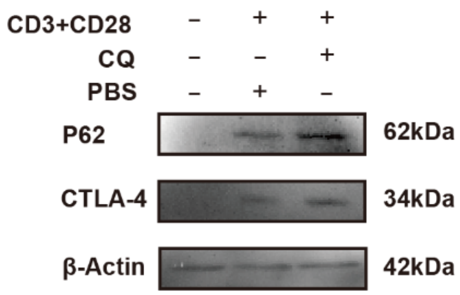

CD3+CD28 – + +
CQ – – +
PBS – + –
P62
62kDa
CTLA-4
34kDa
β-Actin
42kDa

B

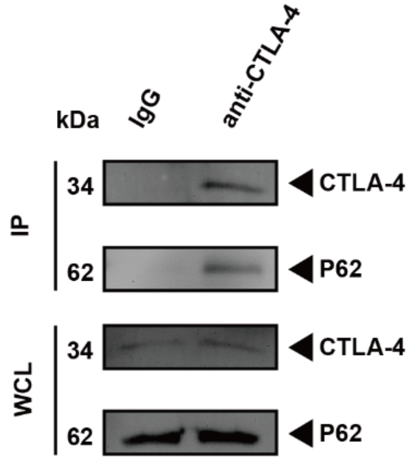

kDa
IgG
anti-CTLA-4
IP
34
CTLA-4
62
P62
WCL
34
CTLA-4
62
P62

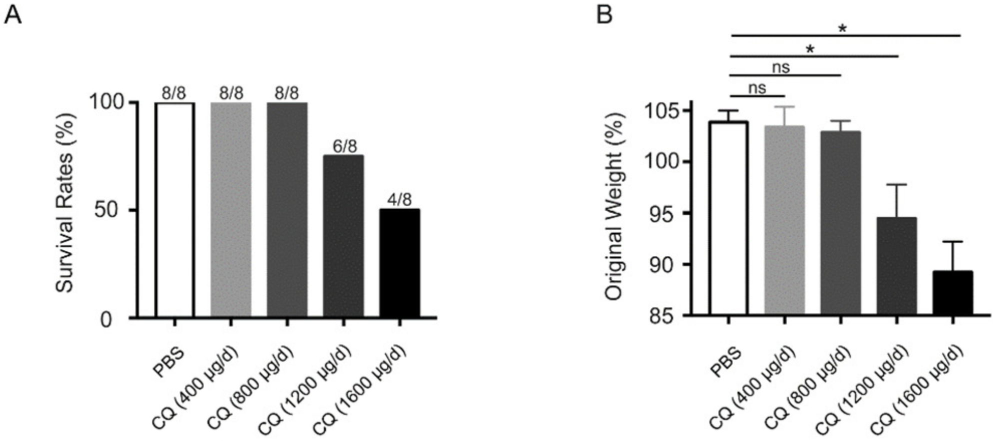
A
Survival Rates (%)
100
50
0
8/8
8/8
8/8
6/8
4/8
PBS
CQ (400 μg/d)
CQ (800 μg/d)
CQ (1200 μg/d)
CQ (1600 μg/d)
B
Original Weight (%)
105
100
95
90
85
*
*
ns
ns
PBS
CQ (400 μg/d)
CQ (800 μg/d)
CQ (1200 μg/d)
CQ (1600 μg/d)

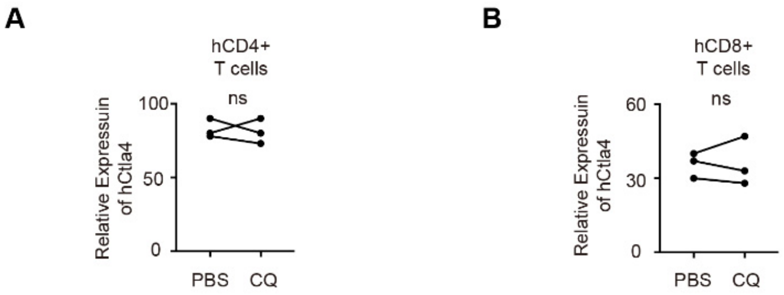
A
hCD4+
T cells
ns
Relative Expressuin of hCtla4
100
50
0
PBS
CQ
B
hCD8+
T cells
ns
Relative Expressuin of hCtla4
60
30
0
PBS
CQ

A

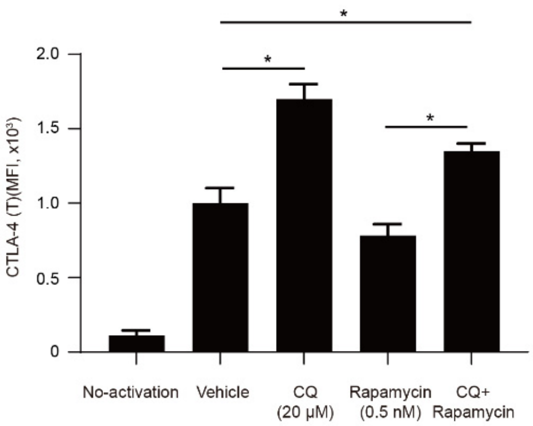

*
*
*
2.0
1.5
1.0
0.5
0
CTLA-4 (T)(MFI, x10³)
No-activation
Vehicle
CQ (20 μM)
Rapamycin (0.5 nM)
CQ+ Rapamycin

B

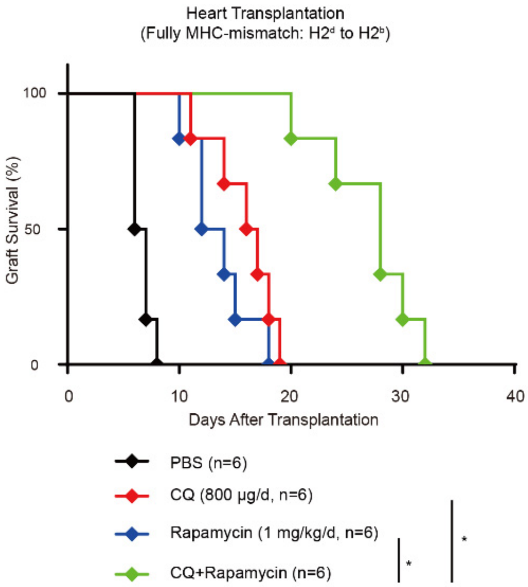

Heart Transplantation
(Fully MHC-mismatch: H2ᵈ to H2ᵇ)
100
50
0
Graft Survival (%)
0
10
20
30
40
Days After Transplantation
PBS (n=6)
CQ (800 μg/d, n=6)
Rapamycin (1 mg/kg/d, n=6)
CQ+Rapamycin (n=6)
*
*
